# The role of probiotics in the treatment of adult atopic dermatitis: a meta-analysis of randomized controlled trials

**DOI:** 10.1186/s41043-022-00318-6

**Published:** 2022-08-17

**Authors:** Menul Ayu Umborowati, Damayanti Damayanti, Sylvia Anggraeni, Anang Endaryanto, Ingrid S. Surono, Isaak Effendy, Cita Rosita Sigit Prakoeswa

**Affiliations:** 1 Doctoral Program of Medical Science, Faculty of Medicine, Universitas Airlangga, Surabaya, Indonesia; 2 Department of Dermatology and Venerology, Faculty of Medicine, Universitas Airlangga - Dr Soetomo General Academic Hospital, Moestopo Number 47, Surabaya, East Java, Indonesia; 3 Department of Pediatrics, Faculty of Medicine, Universitas Airlangga - Dr Soetomo General Academic Hospital, Surabaya, Indonesia; 4 Food Technology Department, Faculty of Engineering, Bina Nusantara University, Jakarta, Indonesia; 5 Dermatology and Allergology, Academic Hospital Bielefeld – Klinikum Bielefeld, Bielefeld, Germany

**Keywords:** Atopic dermatitis, Probiotic, SCORAD, Quality of life, Human and health

## Abstract

**Background:**

Atopic dermatitis (AD) is chronic inflammatory skin disease that is relapsing and a serious condition that disrupts the quality of life of affected individuals. Probiotics are an immunomodulator that can enhance the immune control of atopic dermatitis.

**Methods:**

All randomized controlled trials of probiotics for the treatment of adult AD published before December 2020 were included in this study from the PubMed databases and manual searching.

**Results:**

Six randomized controlled trials (*n* = 241) were selected for this meta-analysis study. Probiotics were effective in treating adult patients with AD, indicated by the decrease in Scoring Atopic Dermatitis/SCORAD (Mean Difference (MD)  − 7.90, 95% CI − 7.25 to − 6.92; *p* < 0.00001; *I*^2^ = 96%) and improved quality of life (MD − 7.68, 95% CI − 14.08 to − 1.29; *p* = 0.02; *I*^2^ = 47%) which were statistically significant. However, skin severity, itch severity, Dermatology Life Quality Index (DLQI), IL-4, TFN-γ, and IgE showed no significant difference in this meta-analysis study (*p* > 0.05).

**Limitations:**

The study found no available data for side effects of probiotics.

**Strength:**

This meta-analysis analyzed a total of 241 AD patients of Asian and European origin.

**Conclusion:**

The use of probiotics decreased SCORAD significantly in adult patients with AD. Probiotics can improve the quality of life of patients with AD.

**Capsule summary:**

The use of probiotics in atopic dermatitis has been widely studied, with controversial results. This meta-analysis suggests that the use of probiotics can improve SCORAD and the quality of life of patients with atopic dermatitis.

## Background

Allergic diseases, including atopic dermatitis (AD), are serious conditions that disrupt the quality of life of affected individuals. AD is a chronic inflammatory skin disease that is relapsing and whose onset is generally related to a patient’s or family’s atopic history such as asthma and allergic rhinitis. This disease is often associated with impaired skin barrier function, allergen sensitization, and recurrent skin infections [1, 2]. Chronic relapse path is a major feature of AD and greatly impacts the quality of life of patients and their families. A study of adolescents with AD showed that patients with mild to moderate disease had a lower quality of life, as measured by the Children's Dermatology Life Quality Index [3, 4].

The onset of AD often occurs at three to six months of age, with nearly 60% of patients experiencing disease progression in the first year of life and up to 90% experiencing such by five years of age [2]. Epidemiological studies have shown that the overall prevalence of AD worldwide is around 1% to 20% [5–7]. A retrospective study found that 7.3% of patients in the dermatologist clinic were affected, with the predominantly afflicted age group being those from 15 to 24 years old (33.3%) [8]. The chief complaint in AD patients who come to the clinic is recurrent itching, dry skin, and redness [9, 10]. Several AD therapeutic approaches have been established, which include promoting skin hydration, emollients, allergen avoidance, and the use of antihistamines or corticosteroids during the exacerbation phase. However, while these therapies can relieve symptoms, their use is often not effective enough, and the recurrence rate is still high [11–15]. Retrospective studies from referral hospital in Indonesia indicate that much of the medication uses mebhydrolin napadisylate and then dexamethasone [16, 17].

A significant amount of information has been released about the use of probiotics in AD but mixed results have been reported, especially in the adult population [18]. Probiotics are widely used as adjuvant therapy for allergic cases with inconsistent results due to the multifactorial mechanism of allergies [19–21]. Prakoeswa et al. in their previous research used a probiotic—namely, *Lactobacillus plantarum* IS-10506, which is isolated from curd as the result of the fermentation of traditional Indonesian buffalo milk—in adult with AD, and reported a significant increase in Foxp3 and interleukin (IL)-10. *Lactobacillus* sp. is important for adjuvant therapy in the treatment of AD and preventing recurrence or development of AD, which plays a role in modulating Th1 and Th2 cytokine profiles. The study showed a decrease in SCORAD index, IL-4, and IL-17 [22]. Another study of *Lactobacillus* sp. for children AD showed similar results in reducing clinical symptoms of AD. That effect was shown by decreasing in SCORAD and levels of serum IgE, IL-4, and IL-17. It decreases the clinical symptoms by suppressing Th2 adaptive immune response, but not increasing the Th1 adaptive immune response [23]. Other research suggested that the probiotic administration decreased the clinical symptoms of AD through the induction of regulatory T-cells (Tregs) and equilibrium of the role of Th1–Th2–Th17 [23–26]. To support probiotics used as adjuvant therapy for allergic cases, a recent study using the same probiotics caused a greater decrease in the Scoring Atopic Dermatitis (SCORAD) index in the group receiving *L. plantarum* compared with the control group [18, 27–29].

Systematic review is needed to establish the highest level of validity of existing researches. Thus, the present systematic review assessed randomized control trials (RCTs) based on the PICO strategy—that is, population = adult with AD; intervention = probiotic intervention; control = standard therapy; and outcome = clinical manifestations (SCORAD evaluation, skin severity, itch severity), quality of life, and/or immune response (i.e., Th1, Th2, Th17, Treg, Th9, and Th22). This study adds more studies than previous reviews by Kim et al. and focuses on adult population.

## Methods

### Literature selection

RCTs published before 1^st^ December, 2020 were sourced from the PubMed databases and manual searching using keywords related to atopic dermatitis and probiotics. In particular, all relevant studies were addressed by using keywords “atopic dermatitis OR eczema” AND “probiotics OR lactobacillus” AND “adult.” The studies must be written in English. We also assessed all the citations of relevant articles manually to supplement this review. Eligible studies included (1) RCTs that involved assessments of living microorganisms in the digestive tract, including bacteria, fungi, or yeast, digested singly or in combination, and (2) participants of adults and any gender who were diagnosed with atopic dermatitis or eczema by a doctor. However, those studies assessing other specific dermatitis such as irritant contact dermatitis were excluded.

### Data extraction, quality assessment, and outcomes

Four independent authors evaluated all the studies retrieved from the databases and reference lists. There was no disagreement, and it was not necessary to add an authorization review for mediation purposes in processing the gathered data. The data collection was tested; then, the gathered data used to summarize each trial’s key points were checked and the data collected. For this review, the following outcomes were extracted from the included studies: changes in the symptoms of dermatitis assessed by SCORAD, skin severity, itch severity, changes in the quality of life reported at the end of therapy, and changes of cytokines (IFN-γ, Serum IgE, IL-4).

### Data analysis

For continuous data, we calculated individual and pooled statistics as mean differences (MD) where studies used the same outcome measure, reported with 95% confidence interval (CI), where possible. Forest plots were created to present the prevalence and the corresponding 95% CI of mean differences and clinical characteristics, respectively. We used *I*^2^ statistics to assess heterogeneity among the studies. *I*^2^ values from 0 to 50% indicate low heterogeneity, *I*^2^ between 50 and 75% indicates moderate heterogeneity, and *I*^2^ more than 75% indicates high heterogeneity. If *I*^2^ < 50%, we used the fixed benefit model to pool the data. Contrarily, when *I*^2^ > 50%, we used the random effect model. The threshold of statistical significance was set to be 0.05. We used a funnel plot to test publication bias. All analyses and plots were performed and created with Review Manager (version 5.3).

## Results

**Fig. 1:**
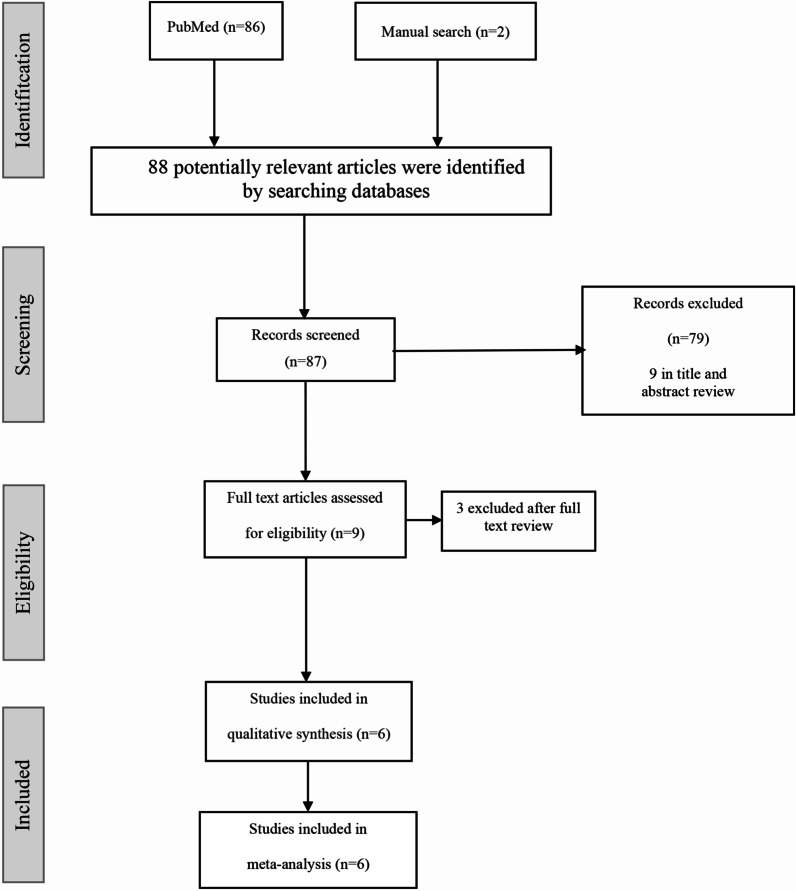
Flowchart of the study search and selection process

This study compiled six studies in total that were identified from the database. Six RCTs involving a total of 241 subjects, including 128 subjects in the probiotics group and 113 subjects in the placebo group, were included in the meta-analysis. Among 241 adult subjects (aged > 14 years old) there were 109 males and 132 females. The studies included in this meta-analysis were conducted in Asian population (Indonesia and Japan) and European population (Italy). The flowchart for the literature search and selection process is shown in Fig. 1. The diagnosis of AD by Hanifin Rajka criteria was used by Prakoeswa et al. and Matsumoto et al., while Moroi et al. used Japanese Dermatological Association criteria for AD. Drago et al. and Iemoli et al. used consensus guidelines in diagnosis and treatment of AD by Eichenfield in 2004 for diagnosing AD. Meanwhile Inoue et al. did not state the criteria for AD used in the study. We analyzed outcomes changes in the symptoms of dermatitis assessed by SCORAD, skin severity, and itch severity, changes in the quality of life reported at the end of therapy, and changes of cytokines (IFN-γ, Serum IgE, IL-4). A tabulation of study author(s), publication date, research design, treatment approach, recruited numbers, and patient age was prepared from the included studies (Table 1).

**Table 1:** Characteristics of studies included in the review

Trial	Methods	Participant		Intervention (*n*)	Comparison (*n*)	Primary outcome	Secondary outcome
Prakoeswa, 2020 (Indonesia)	DB, RCT	30 adult patients with mild to moderate AD (aged > 14 years), serum IgE levels > 100 IU/ml		Probiotic microencapsulation of *Lactobacillus plantarum* (LPIS-10506) 2 × 10^10^ CFU per day for 8 weeks (*n* = 15, 4 males, 11 females, mean age 37.67 ± 15.92)	Placebo group with skim milk-Avicel (*n* = 15, 5 males, 10 females, mean age 38.07 ± 12.84)	SCORAD IgE IL-17 IFN-γ FOXP3 +	Skin lesion area DLQI Safety assessment (adverse drug reaction)
Drago, 2011 (Italy)	DB, RCT	38 patients (18 males and 20 females) aged 18–46 years old with moderate/severe AD		Probiotic *Lactobacillus salivarius* (LS01) 1 × 10^9^ CFU/g in maltodextrin, twice daily for 16 weeks (*n* = 19, mean age 32.07 ± 1.79)	Placebo group with maltodextrin (*n* = 19, mean age 28.86 ± 2.15)	SCORAD DLQ Serum IgE IL-12 IFN-γ IL-4 IL-5	-
Iemoli, 2012 (Italy)	DB, RCT	48 adult AD patients, 20 males and 28 females. 2 patients were lost to follow-up and excluded (1 in each group)		*Lactobacillus salivarius* (LS01 DSM 2275) and *Bifidobacterium breve* (BR03 DSM 11,604), each with dose of 1 × 10^9^ CFU/g in maltodextrin, twice daily for 12 weeks (*n* = 31, mean age 32.44 ± 1.47)	Placebo group with maltodextrin (*n* = 15, mean age 30.91 ± 2.79)	SCORAD DLQ Plasma LPS Treg Active T-lymphocyte	
Inoue, 2015 (Japan)	DB, RCT	49 patients with AD (> 16 years old)		*Lactobacillus acidophilus* (L-92) 20.7 mg/day in tablet, for 8 weeks (*n* = 24, 14 males, 10 females, mean age 29.6 ± 13.8)	Placebo group with tablet containing maltose, starch, vegetable oil and fat (*n* = 25, 14 males, 11 females, mean age 29.7 ± 14.5)	SCORAD Serum IgE Eosinophil LDH TALC IL-4, IL-5. IL-6, IL-10, IL-12p70, IL-13, IL-17, IL-18 Eotaxin IFN-γ TNF-α TGF-β	
Moroi, 2010 (Japan)	DB, RCT	34 adult patients with mild to moderate AD (aged 20–65 years old)		*Lactobacillus paracasei* (K71) 5 × 100 mg (~ 2 × 10^11^ bacteria) and 400 mg dextrin NSD300 in powder, daily for 12 weeks (*n* = 17, 5 males, 12 females, mean age 29.4 ± 5.7)	Placebo group with 500 mg dextrin and 0.45 mg carotene base in powder (*n* = 17, 5 males, 12 females, mean age 31.6 ± 10.1)	Skin severity score Itch score QoL impairment score	
Matsumoto, 2014 (Japan)	DB, RCT	44 adult patients with moderate to severe AD		*Bifidobacterium animalis* subsp. Lactis (LKM 512) 6 × 10^9^ CFU with excipients (skim milk, glucose, inulin, dextrin, silicon dioxide) in capsule, daily for 8 weeks (*n* = 22, 14 males, 8 females, mean age 33.5 ± 8.6)	Placebo group with only excipients in capsule (*n* = 22, 10 males, 12 females, mean age 34.1 ± 8.7)	Itch improvement level VAS score QoL Fecal microbiota	

*RCT* randomized controlled trial, *DB* Double blind

### Clinical effects by the severity of AD

**Fig. 2:**
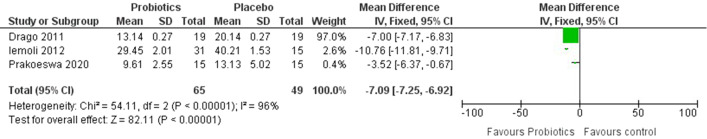
Forest plot of SCORAD values stratified by disease severity

**Fig. 3:**
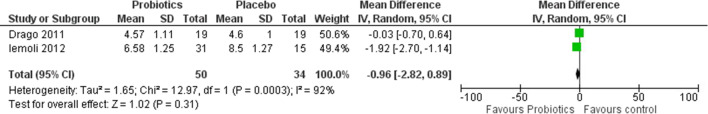
Forest plot of the DLQI

**Fig. 4:**
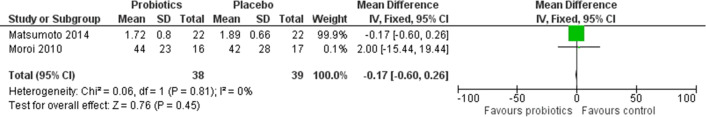
Forest plot of the itch severity

**Fig. 5:**
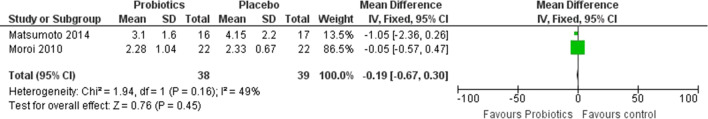
Forest plot of the skin severity score

Probiotics were effective in treating subjects with moderate AD in adult and decreased SCORAD significantly (MD − 7.90, 95% CI − 7.25 to − 6.92; *p* < 0.00001; *I*^2^ = 96%) in three studies (Fig. 2). However, skin severity and itch severity showed no significant difference (MD − 0.17, 95% CI − 0.60 to 0.26; *p* < 0.45; *I*^2^ = 0% and MD − 0.19, 95% CI − 0.67 to 0.30; *p* < 0.45; *I*^2^ = 49%) in two studies included (Figs. 3, 4, 5).

### Quality of life index

The assessment for quality of life was conducted in four out of six studies reviewed in this meta-analysis. Prakoeswa et al., Iemoli et al., and Drago et al. used Dermatology Life Quality Index (DLQI) as a tool to assess the life quality of the patients, but Prakoeswa et al. assessed the DLQI as secondary outcome without mentioning the DLQI score. Meanwhile, Moroi et al. and Matsumoto et al. used Skindex-16 and Skindex-29 to determine the quality of life, respectively. A random-effects model meta-analysis of two studies reported the quality index of 84 total subjects, including 50 subjects in the probiotics group and 34 subjects in the placebo group (Fig. 3). The analysis of data showed that the DLQI was not improved significantly in the probiotics group (MD − 0.96, 95% CI − 2.82 to 0.89; *p* = 0.31; *I*^2^ = 92%).

### Cytokines

**Fig. 6:**
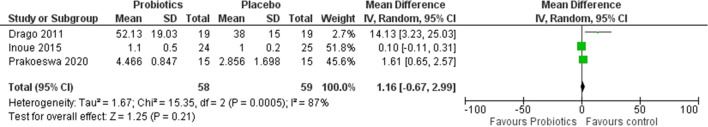
Forest plot of IFN-γ

**Fig. 7:**
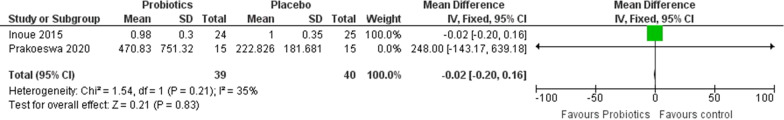
Forest plot of the serum IgE

**Fig. 8:**
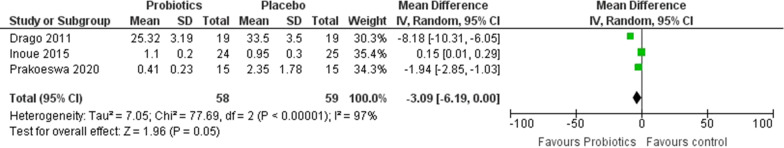
Forest plot of the IL-4

There were three studies, including a total of 117 subjects, that reported the results of cytokines IL-4 and interferon (IFN)-γ. The meta-analysis revealed no difference existed regarding the IL-4 and (IFN)-γ level in the probiotics group and the placebo group difference (MD − 3.09, 95% CI − 6.19 to 0.00; *p* = 0.05; *I*^2^ = 97% and MD 1.16, 95% CI − 0.67 to 2.99; *p* < 0.21; *I*^2^ = 87%) (Figs. 6, 7). Other result of cytokines is serum IgE, which, in this meta-analysis, showed no significant difference (MD − 0.02, 95% CI − 0.20 to 0.16; *p* = 0.83; *I*^2^ = 35%) (Fig. 8). Finally, we conducted a pooled analysis and critical appraisal regarding the effect of probiotics on AD and found that, in this meta-analysis, all of the included studies were valid, important, and applicable.

## Discussion

There is increasing evidence that the intestinal microbiome plays an important role in modulating systemic inflammation and disease. While the exact nature of the gut microbiome naturally varies between individuals, there can also be fluctuations attributable to myriad exogenous and endogenous factors. Factors that result in a negative biome balance predispose the host to offering an environment in which virulent bacterial strains, such as *Escherichia coli*, *Pseudomonas aeruginosa*, and *Enterococcus faecalis*, come to dominate the gastrointestinal tract [30].

The utility of oral probiotics for the treatment and prevention of AD has been explored through several large cohorts and randomized controlled studies. In this recent meta-analysis, including 241 patients, we analyzed some outcomes: clinical effect (SCORAD values, skin severity, itch severity), quality of life (QOL), and cytokines (serum IgE, TNF-γ and IL-4) for patients receiving oral probiotics.

### Clinical effect

The preventive and therapeutic actions of probiotics in the intestine in patients with AD are of key consideration. Prior studies have suggested that childhood and adult enteric infection and bacterial exposure in the gastrointestinal tract may protect individuals from allergies [31, 32]. From the included studies, we found that probiotic supplementation reduced the clinical manifestations of AD. A mixture of seven probiotic strains and fructooligosaccharides may clinically improve the severity of AD in adults. Considering the numerous beneficial effects of probiotics, their simple administration route, and their low side effects, assessing the effect of these treatments on other allergies such as food allergies, asthma, and allergic rhinitis, is an area for future research [33].

Clinical improvement in AD was assessed by changes in disease severity. AD disease severity can be assessed by a variety of methods, the most widely used being the SCORAD score. The SCORAD index assesses the intensity of skin lesions (erythema, papules, crusts, excoriation, lichenification, and dryness) which is then formulated into a score. The higher the score, the worse the disease. In this meta-analysis study, three of the six studies, including Prakoeswa et al., Drago et al., and Iemoli et al., used SCORAD as a parameter of clinical improvement.

Specific mixture of probiotics (LS01 and BR03 strains) may induce beneficial effects with respect to clinical and immunologic alterations in adult AD. This combination could be considered as adjuvant therapy for the treatment of AD in adult patients [34]. Mixture of probiotics (*Bifidobacterium lactis* CECT 8145, *B longum* CECT 7347, and *Lactobacillus casei* CECT 9104) was effective in reducing SCORAD values and the use of topical steroids in patients with moderate AD [32]. Karim et al. showed significantly reduced SCORAD in the fourth week and eighth week after treatment with probiotics [35]. Finally, Gore et al. found no benefit from supplementation with *B. lactis* or *Lactobacillus paracasei* in the treatment of eczema when given as an adjunct to basic topical treatment and no effect on the progression of allergic disease from one to three years of age [36–39]. Iemoli et al. assessed the clinical efficacy of an intake of a combination of two probiotics (*Lactobacillus salivarius* LS01 and *Bifidobacterium breve* BR03) for the treatment of adult AD patients. Patients receiving probiotics showed a significant reduction in SCORAD value at the end of treatment, which persisted after suspension, and an improvement in DLQI [34]. Some previous studies, randomized, double-blind, and placebo controlled study, evaluated the clinical symptoms showed significantly reduced SCORAD in adult AD after probiotic treatment [28, 34]. Drago et al., in the probiotic group, showed significantly reduced SCORAD after four months treatment; however, Prakoeswa et al. showed the result after eight weeks treatment. Decrease in SCORAD was not associated with total IgE level [22, 40]. Clinical symptoms also showed in skin severity index after probiotic treatment in adult AD. Some studies suggested that the skin severity index can be reduced after eight weeks of treatment [41, 42].

### Immunological response

Probiotics demonstrate an immunomodulatory effect and are able to improve intestinal barrier function and reduce the inflammatory reaction in allergy diseases such as AD by inhibiting the epithelial and mucosal adherence of pathogens and preventing pathogen invasion through the epithelium [33, 43, 44]. Probiotics restore the mucosal barrier function in the intestines and degrade food antigens, which may decrease the rate of pathogen proliferation [31, 33, 45].

Th2 cells are more predominant than Th1 cells in AD, which causes an imbalance between these two cell groups, leading to defects in filaggrin and resulting in skin-barrier dysfunction and decreased protection from pathogens and allergens. There has been speculation that exposure to microbial agents at an early age may increase the rate of Th1 cell maturation and decrease the Th2 cell response. Prebiotics increase the induction of Tregs and modulate Toll-like receptors (TLR), which can activate dendritic cells and the Th1 response or may cause direct induction on T-cells [31, 33, 46]. Other studies reported that probiotics increase Th1 function and decrease Th2 and Th17 activity. Th17 cells are increased in acute AD and correlate with disease severity [34, 44].

The present meta-analysis revealed that there was no difference in IL-4 levels between the probiotics group and the placebo group. Meanwhile, IFN-γ (*p* = 0.45) was not statistically significantly decreased in the placebo group [31]. Some studies showed significant modulation of the innate and adaptive immune response except IL-4 and total IgE [47]. Bonita et al. showed no significant difference of IgE after treatment with probiotics [48]. Farid's study showed that there was no significant difference between the probiotic and placebo groups with respect to baseline characteristics. However, no specific effect of the probiotics employed was demonstrated on the IL-4 and IFN-γ (*p* = 0.05 and *p* = 0.21) [33, 36, 37]. Treg decreased in AD patients while IgE, eosinophilia, and IFN- γ levels increased. Probiotics species can induce Treg [49, 50]. Some studies findings showed that *Lactobacillus* species can induce Treg stimulation by signaling pathway mediated by dendritic cell-specific intracellular adhesion molecule 3-grabbing nonintegrin (DC-SIGN) [51].

Probiotics play a role of improving gut permeability in AD patients. This can be seen in the restoration of the intestinal protective function. Several studies have shown a significant reduction in microbial translocation after probiotic treatment in AD patients [51]. Iemoli et al. stated that the combination of probiotics greatly influences immunomodulatory activity as the ability to increase barrier function [34]. In addition, probiotic strain has important role in modulating Th1 and Th2 cytokine profile. Therefore, probiotics can be used as adjuvant treatment of adult AD.

### Quality of life

Atopic dermatitis causes chronic itching and scratching, which might impact the patient’s psychosocial and quality of life (QOL). Decreased QOL has been linked with sleep deprivation and depressive symptoms and may have a possible effect of AD treatments. Chronic sleep loss in AD patients contributes to emotional and physical fatigue that negatively impacts existing social sensitivity and social relationships. Other data showed that sleep quality was inversely associated with disease severity in AD patients [52, 53]. Sleep loss also can increase IL-6 production in AD patients, which is involved in the regulation of the immune system. That correlation may also partially reflect circadian rhythm patterns known to be associated with itch mediators [54]. The analysis of data showed that the QOL improved significantly in the probiotics group. The studies in this meta-analysis evaluated the QOL by using DLQI, Skindex-16 and Skindex-29. Iemoli et al. and Drago et al. measured the improvement of AD symptoms with DLQI questionnaire. The questionnaire consisted of 10 questions and was structured with corresponding scores of 0, 1, 2, 3 and 4. Improvement of quality of life is indicated by high score. DLQI was evaluated at 0, 12th, and 20th weeks [34, 40]. Matsumoto et al. examined the effects of the probiotic *Bifidobacterium animalis* subsp. *lactis* LKM512 on adult-type AD and the expression of metabolites that are known to be influenced by gut microbiota in fecal samples. Matsumoto et al. assessed the dermatology-specific quality of life by using the Skindex-29, developed by Chren et al. [36] which consists of 30 items forming three scales (item 18 was not included in scoring), including emotion (10 items), symptoms in (seven items), and functioning (12 items). Not only in adult patients, the use of probiotics has also been shown to improve the QOL in children with AD, as found in the Wang et al. report [36–38].

The microbiome serves as a possible treatment target and modulating it using probiotics is one way. While rapid increases in the medical use of probiotics have verified their excellent safety profile, long-term safety data are limited. Of concern, reports also link probiotics to infections and other severe side effects in immunocompromised individuals. Thus, more basic research and epidemiological studies are required to characterize further the microbiome as a risk factor and in the treatment of disease [55, 56]. Additionally, future probiotic trials could yield interesting results to support a microbiome replacement strategy.

## Conclusion

AD can affect a patient's psychological condition and quality of life, which can lead to depression. This meta-analysis study showed that probiotic supplementation may have the potential to decrease disease severity (SCORAD) in adult AD. The decrease in disease severity may also improve the quality of life. Therefore, probiotics can be used as adjuvant treatment of adult AD**.**

## Data Availability

Not applicable.

## Abbreviations

AD: Atopic dermatitis
CD: Cluster of differentiation
CI: Confidence interval
DQoL: Dermatitis Quality of Life
IFN: Interferon
IL: Interleukin
MD: Mean difference
SCORAD: Scoring Atopic Dermatitis
